# Effectiveness of a mRNA Vaccine Booster Dose Against COVID-19 Among Oregon Healthcare Personnel, January 2021-June 2023

**DOI:** 10.1017/ash.2025.300

**Published:** 2025-09-24

**Authors:** Madysen Schreiber, Eric Hall, Valerie Ocampo, Alexia Zhang, Angela Dusko

**Affiliations:** 1 Oregon Health & Science University-Portland State University School of Public Health; 2 Oregon Health Authority

## Abstract

**Background:** The prioritization of U.S. health care personnel (HCP) for early receipt of messenger RNA (mRNA) vaccines against severe acute respiratory syndrome coronavirus 2 (SARS-CoV-2) allowed for the evaluation of the effectiveness of these vaccines in a real-world setting among a high-risk population. The purpose of this study was to summarize the sociodemographic characteristics of HCP in Oregon eligible to receive COVID-19 vaccination and estimate vaccine effectiveness (VE) of a mRNA COVID-19 vaccine booster dose. **Methods:** We conducted a case-control study involving HCP from 5 hospitals in Oregon. Cases were defined as those with a positive antigen test or nucleic acid amplification test (NAAT) for SARS-CoV-2. Controls were defined as those with a negative antigen test or NAAT result and were matched to cases by site and within a 2-week interval of test date. Using conditional logistic regression with adjustment for age, sex, race and ethnicity, educational level, underlying conditions, and reported exposure to COVID-19, we estimated VE for a 3rd COVID-19 vaccine booster dose (with the 3rd dose more than 150 days after the 2nd dose). VE was estimated using the screening method as 1-odds ratio x 100%. **Results:** Among 865 HCP, 374 (43%) were included as case-participants and 491 (57%) as control-participants. Overall, the adjusted VE of a booster dose was 62.6% (95% CI: 37.6%, 77.6%), compared to vaccination with 2 mRNA doses. Logistic regression analysis indicated that HCP with a college degree (vs. no degree, OR: 3.26, 95% CI: 1.96, 5.45), private insurance (vs. government/military, OR: 3.32, 95% CI: 1.28, 8.59), and an income level $200K+ (vs. <$50K, OR: 3.25, 95% CI: 1.60, 6.60) were more likely to have received the booster vaccine. **Conclusions:** The mRNA COVID-19 booster vaccines conferred approximately 63% protection against COVID-19 among Oregon HCP and were found to be effective under real-world conditions. These findings indicate moderate initial protection against SARS-CoV-2 infection and encourage remaining up-to-date with subsequent COVID-19 vaccines. The identification of sociodemographic characteristics of HCP who are more likely to have received a booster vaccine provides insight into those at higher risk for adverse COVID-19 outcomes due to lower vaccine coverage. While understanding these characteristics is valuable for directing ongoing vaccination efforts towards these populations, further research is needed to understand the mechanisms that contribute to variations in vaccine uptake.

